# Approaches to Assure Similarity between Pharmaceutical Heparins from Two Different Manufacturers

**DOI:** 10.3390/pharmaceutics15041115

**Published:** 2023-03-31

**Authors:** Francisco Felipe Bezerra, Stephan N.M.C.G. Oliveira, Rodrigo A. Sales, Adriana A. Piquet, Nina V. Capillé, Eduardo Vilanova, Ana M.F. Tovar, Paulo A.S. Mourão

**Affiliations:** Laboratório de Tecido Conjuntivo, Hospital Universitário Clementino Fraga Filho and Instituto de Bioquímica Médica Leopoldo de Meis, Universidade Federal do Rio de Janeiro, Rio de Janeiro 21941-913, Brazil

**Keywords:** interchangeable drugs, anticoagulant, NMR analysis, structure of heparin, glycosaminoglycans

## Abstract

Pharmaceutical heparins from different manufacturers may present heterogeneities due to particular extraction and purification procedures or even variations in the raw material manipulation. Heparins obtained from different tissues also differ in their structure and activity. Nevertheless, there is an increased demand for more accurate assessments to ensure the similarities of pharmaceutical heparins. We propose an approach to accurately assess the similarity of these pharmaceutical preparations based on well-defined criteria, which are verified with a variety of refined analytical methods. We evaluate six commercial batches from two different manufacturers which were formulated with Brazilian or Chinese active pharmaceutical ingredients. Biochemical and spectroscopic methods and analysis based on digestion with heparinases were employed to evaluate the purity and structure of the heparins. Specific assays were employed to evaluate the biological activity. We observed minor but significant differences between the constitutive units of the heparins from these two manufacturers, such as the content of *N*-acetylated α-glucosamine. They also have minor differences in their molecular masses. These physicochemical differences have no impact on the anticoagulant activity but can indicate particularities on their manufacturing processes. The protocol we propose here for analyzing the similarity of unfractionated heparins is analogous to those successfully employed to compare low-molecular-weight heparins.

## 1. Introduction

**Figure 1:**
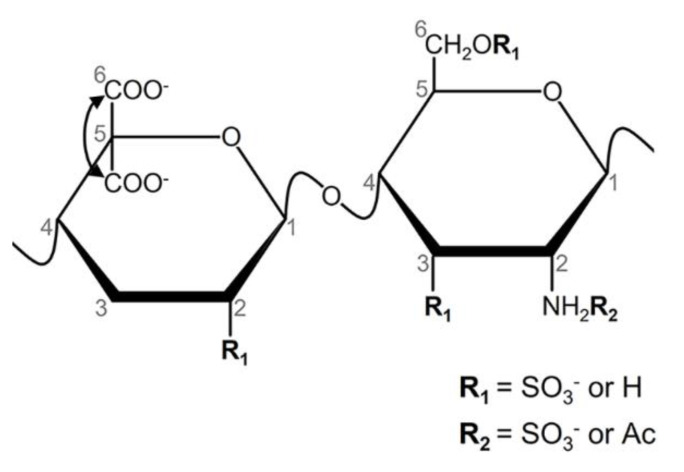
Structure of heparin. This glycosaminoglycan contains alternating units of 2–sulfated α-iduronic acid and *N*,6–disulfated α–glucosamine with several minor modifications (R_1_ and R_2_). We highlight the occurrence of *N*–acetylated α-glucosamine and residues of non-sulfated α-iduronic acid or substituted with β–glucuronic acid. Minor units of *N*,3,6–trisulfated α-glucosamine have an impact on the anticoagulant activity. Protons/carbons are enumerated on the pyranose rings and C6.

Heparin is a glycosaminoglycan mainly composed of alternating units of 2-sulfated α-iduronic acid (α-IdoA2S) and *N*,6-disulfated α-glucosamine (α-Glc*N*,6-diS); however, with several minor structural modifications. Units of *N*-acetylated α-glucosamine (α-Glc*N*Ac) and residues of non-sulfated α-iduronic acid (α-IdoA) or substituted by β-glucuronic acid (β-GlcA) can also occur [1]. Moreover, minor components, such as *N*,3,6-trisulfated α-glucosamine (α-Glc*N*,3,6-triS), have a direct impact on anticoagulant activity [2] (Figure 1).

Unfractionated heparin has been massively used as the main anticoagulant drug for over a century [3]. Even nowadays, when their low-molecular-weight derivatives (LMWHs) and the direct oral anticoagulants are increasingly prescribed for the treatment and prophylaxis of thromboembolic diseases, UFH remains the most potent intravenous anticoagulant, indispensable for patients requiring a rapid, reversible and stable low-coagulant state, such as those undergoing surgeries with cardiopulmonary bypass, extracorporeal membrane oxygenation (ECMO), and hemodialysis [4]. Such a remarkable anticoagulant activity of heparin is promoted by the inhibition of different proteases of the coagulation cascade, especially activated thrombin (FIIa) and activated factor X (FXa) through the potentiation of antithrombin triggered by a specific pentasaccharide epitope [5]. Although currently employed as anticoagulant/antithrombotic drug only, several preclinical and clinical studies have already demonstrated that heparin has therapeutical potential for threatening other diseases, such as atherosclerosis, inflammations, cancer, and viral infections [6]. In fact, heparins were massively employed for the treatment of lung coagulopathies of COVID-19 patients [7].

Despite advances in biotechnology, heparin is still exclusively obtained from animal tissues [8]. Until the end of the 1960s decade, it was extracted from bovine lung and then gradually prepared from porcine intestine. Pharmaceutical preparations of heparin obtained from these two animal sources were marketed interchangeably during such transition period [9]. Heparin from bovine intestine has also been interchangeably used with porcine heparin, mainly in Latin American countries [10]. Only recently were these two types of pharmaceutical preparations differentiated in Brazil, each one with a specific pharmacopeia compendial regulation [11].

During the initial period of the clinical use of heparin, pharmaceutical preparations were evaluated by rudimentary biochemical methods and clotting assays. Notwithstanding refined methods have become routinely available in the last two decades, analysis of heparin is still challenging due to its heterogeneous nature; for example, minor components with a significant impact on anticoagulant activity require laborious methods for identification [12]. The demonstration of similarity among pharmaceutical heparins is far more complex than it is for proteins, which have routine methods for assuring their amino acid sequence and conformation [13]. The heterogeneity of pharmaceutical heparins can arise from differences in extraction and purification procedures or even variations in the animal raw-material preparation. Furthermore, heparins extracted from different tissues also have specific characteristics [14]. Consequently, we need refined analytical procedures to assure the similarities of heparin preparations, as well as identify the tissue of origin.

Three events contributed for increasing the demand for more refined analyses of pharmaceutical heparins. One of them was the contamination with oversulfated chondroitin sulfate, which induces severe side effects in patients. Later, it was demonstrated that even minor amounts of that toxic contaminant can in fact cause serious adverse effects [15,16,17,18]. Another event was the availability of refined and automated analytical methods, which can now be routinely used to assess pharmaceutical quality. Finally, medical practice is increasingly demanding better drugs, which ensure high effectiveness and safety. Heparin is often prescribed for critically ill patients who require strictly controlled medications [19]. Nevertheless, there is an unmet demand for more accurate assessments to ensure the similarity of pharmaceutical heparins. This aspect has already been established for low-molecular-weight heparins, with guidelines for comparative studies published by regulatory agencies [20,21]. However, unfractionated heparin, which has many particularities compared to low-molecular-weight heparins, still requires protocols for similarity assessments.

In this study, we evaluated six batches of commercial heparin from two Brazilian pharma companies, one formulated with active pharmaceutical ingredient (APIs) from China and another from Brazil. These preparations were assessed according to criteria and analytical methods described in Table 1, which in turn combines the compendial methods with more refined procedures for evaluating pharmaceutical heparins. Such a variety of methods enables the assessment of the physicochemical properties and biological activity by a combination of analytical approaches; in particular, we combined spectroscopic methods and analysis based on digestion with specific enzymes to evaluated the disaccharide composition of heparin. Our objectives are: (1) To assess the differences between heparins obtained from two manufacturers and among their batches; (2) To evaluate the impact of minor physicochemical differences on the biological activity; and (3) To establish the approaches to ensure precisely the similarity of pharmaceutical heparins.

## 2. Material and Methods

### 2.1. Heparins and Standards

This study was conducted with pharmaceutical heparins, available in ampules containing 5000 IUs dissolved in 0.25 mL of water for injection. These preparations, commercially available for subcutaneous administration, were obtained from two Brazilian pharma companies (three batches of each). They were formulated with APIs produced in Brazil (Manufacturer 1) or China (Manufacturer 2). For spectroscopic analysis and assays based on heparinases digestions, these solutions were lyophilized to obtain the dry powder. The 6th International Heparin Standard (2145 units per vial, Lot No. 07/328) and 2nd International Standard of Low-Molecular-Weight Heparin for molecular weight calibration (coded 05/112) were from the National Institute for Biological Standards and Control (NIBSC; Potters Bar, United Kingdom), Heparin sodium molecular-weight calibrant (Lot No. FOL4830) was from USP (Rockville, MA, USA). Standards of dermatan sulfate and oversulfated chondroitin sulfate (Lot No. HOM191) were purchased from Sigma-Aldrich (St. Louis, MO, USA) and USP, respectively.

### 2.2. Biochemical Assays

Concentrations of heparin on the pharmaceutical batches were estimated by carbazole reaction, using as standard a sodium heparin from USP [22]. The content of protein was quantified with the bicinchoninic acid method, using bovine-serum albumin (Sigma) as standard [23]. Analysis of nucleic acid content was based on absorbance at 260 nm. Agarose gel electrophoresis was performed as described elsewhere [24].

### 2.3. Ion-Exchange Chromatography

Samples (200 µg) of the pharmaceutical heparins from two manufacturers and a mixture containing sodium heparin standard (200 µg), dermatan sulfate (30 µg) and oversulfated chondroitin sulfate (20 µg) were applied into a Dionex Ion Pac AS11-HC polymer column (250 mm high × 2 mm internal diameter), coupled to a HPLC system (Shimadzu). The mobile phase used for eluting the samples resulted from mixing 20 mM Tris-HCl (pH 7.4) (Solution A) with the same solution containing 2.5 M NaCl (Solution B), according to the gradient method previously described [10]. The columns were eluted with a flow of 0.5 mL min^−1^ and continuously monitored by UV absorbance (232 nm), subtracted from the respective background. The heparin samples were also treated with nitrous acid (100 µL samples containing 1 mg glycosaminoglycan, 50 µL of 2 M sodium nitrite and 50 µL of 1 N H_2_SO_4_). After incubation for 1 h at room temperature, the reaction was stopped by adding 50 µL of 1 M NaOH. The reaction products were analyzed with ion exchange chromatography, as described above. In order to check the homogeneity of the heparins, 5 mg of the pharmaceutical heparins and a pharmaceutical batch of bovine heparin were applied into a Fractogel EMD TMAE Hicap (Sigma-Aldrich), linked to a HPLC system (Shimadzu), equilibrated with 20 mM Tris-HCl, 1 mM EDTA (pH 7.4). Heparins were eluted with a flow rate of 1.0 mL min−1 through a step-wise gradient of an equilibration buffer supplemented with 16.7% (5 min) →60% (20 min) →100% (10 min) 2 M NaCl continuously monitored by UV absorbance (215 nM).

### 2.4. Size-Exclusion Chromatography

Heparins from two manufacturers and the “Sodium heparin molecular weight calibrator” from USP (100 µg of each) were applied into a set of columns TSK G-4000 and TSK G-3000 (Tosoh), coupled to an HPLC system (Shimadzu). The samples were eluted at 0.3 mL min^−1^ with 0.1 M ammonium acetate (pH 6.0) and continuously monitored by refractive index (RID). The data acquired with the USP calibrator were expressed as the log of molecular mass vs. elution time (min) and the results were fitted on a third-order polynomial equation. The parameters obtained with the USP calibrator were used to calculate the profile of molecular mass distribution, as previously described [10].

### 2.5. NMR Spectrometry

NMR spectra of the pharmaceutical heparins were recorded using a DRX 800 MHz Spectrometer (Bruker; Billerica, MA, USA) with a triple-resonance probe, as described [10]. Approximately 20 mg of each heparin batch were dissolved in 0.5 mL 99.9% deuterium oxide (Cambridge Isotope Laboratory; Cambridge, MA, USA) and then the spectra were recorded at 35 °C with HOD (deuterium oxide) suppression by pre-saturation. One-dimensional ^1^H NMR spectra were recorded with 32 scans. Phase-sensitive ^1^H-^1^H MLEV17 TOCSY spectra (4046 × 400 points) were acquired with spin-lock field of 10 kHz and mix time of 80 ms. ^1^H-^13^C multiplicity-edited HSQC spectra (1024 × 256 points) were acquired with globally optimized alternating phase rectangular pulses for decoupling (GARP). ^1^H and ^13^C chemical shifts were calibrated (0 ppm) with basis on signals from external standards trimethylsilyl propionic acid and methanol (both from Sigma-Aldrich), respectively. Spectra were processed using the software Top-Spin 4.0 (Bruker).

### 2.6. Disaccharide Analyses with SAX-HPLC Chromatography

Heparin disaccharides were produced by incubating 10 mg mL^−1^ of heparin and 4.0 Sigma units mL^−1^ each of heparinases I, II, and III (all from *Flavobacterium heparinum*; Sigma-Aldrich) in 20 mM Tris HCl for 24 h at 37 °C. The products of these incubations were diluted with distilled water 1:8, 20 µL of each and a mixture of standards of heparin disaccharides were applied into a SUPELCOSIL SAX1 column (Sigma-Aldrich) coupled to an HPLC system (Shimadzu). The disaccharides were eluted at 0.2 mL min^−1^ through a gradient of 0→1.0 M NaCl in deionized water, containing 0.1 M HCl, and continuously monitored by absorbance at 232 nm. The retention times and peak integrals of the disaccharides were calculated using the HPLC software Shimadzu LC Solution Rel. 1.25 (Shimadzu).

### 2.7. Oligosaccharide Analyses with Size-Exclusion Chromatography

Oligosaccharides were produced by incubating samples (200 µL) containing 5 mg of each heparin batch with 10 µL of heparinase I (250 U mL^−1^, Sigma-Aldrich) in 20 mM Tris HCl for 24 h at 37 °C. The incubations’ products were applied into TSK G-3000 + TSK G-2000 (Tosoh) columns, coupled in series to an HPLC system (Shimatzu), and then eluted at 0.3 mL min^−1^ with 0.1 M ammonium acetate (pH 6.0). Fractions were continuously monitored by refractive index. In parallel, the standards of unfractionated and of low-molecular-weight heparins were also analyzed. Retention times and peak integrals of the oligosaccharides were determined using the HPLC software (Shimadzu).

### 2.8. Fluorescence Spectroscopy to Determine Heparin–Antithrombin Interactions

Affinities of the pharmaceutical heparins with antithrombin were evaluated based on dissociation constants (*K*_D_), calculated through the fluorescence gain of the serpin promoted by the binding to heparin, as previously described [25]. Changes in the intrinsic fluorescence of antithrombin were monitored from 300 to 450 nm, with excitation wavelength set to 280 nm, using a Varian Cary Eclipse spectrofluorometer (Varian, Palo Alto, CA, USA). All the spectra were acquired at 37 °C under continuous stirring and the bandwidths set to 5 nm for excitation and 10 nm for emission. Dissociation constants (*K*_D_) for the heparin–antithrombin bindings were calculated on the basis of enhancements in tryptophan fluorescence emissions by nonlinear regression, as described [25]. Spectral areas were calculated using Cary Eclipse Softwar v. 1.1(132) (Varian).

### 2.9. Activated Partial Thromboplastin Time Assay (APTT)

The anticoagulant activity of the solutions of pharmaceutical heparins were determined by the APTT assay, using human plasma, as described [10]. The clotting time was recorded in an Amelung KC4A coagulometer (Heinrich Amelung GmbH; Lemgo, Germany). The results were expressed as the ratio of clotting time in the presence and absence of different volumes of the heparin solutions and then fitted as second*-*order polynomial curves. Anticoagulant potencies in IU mL^−1^ were calculated on the basis of values obtained fitting the 6th International Heparin Standard curve [10]. Anticoagulant potencies were compared by *t*-test using Origin 8.0 software (OriginLab; Northampton, MA, USA).

### 2.10. Anti-FXa and Anti-FIIa Activities

These activities were determined based on FIIa or FXa amidolytic activity assessed by measuring the hydrolysis of chromogenic substrates, as described in [10]. Anti-FXa and anti-FIIa activities were calculated on the basis of parallel line assays performed with the 6th International Heparin Standard using SoftMax Pro 5.4.1 software (American Devices). The anti-FXa and -FIIa activities of heparins from the two manufacturers were compared by *t*-test using Origin 8.0 software (OriginLab).

## 3. Results and Discussion

### 3.1. Biochemical Methods

**Figure 2:**
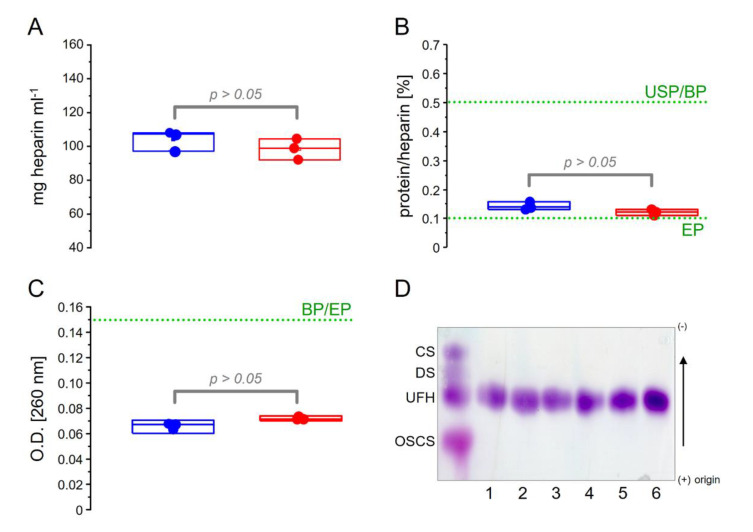
Hexuronic acid (**A**), protein (**B**), and nucleic acid (**C**) contents of the three batches of heparin from each manufacturer 1 (**blue**) and manufacturer 2 (**red**). The broken lines in green indicate the limits accepted by the US (USP), Brazilian (BP), and European (EP) Pharmacopeias. Panel (**D**) shows the agarose gel electrophoresis of the heparins stained with toluidine blue. Electrophoretic migration of chondroitin 4–sulfate (CS), dermatan sulfate (DS), sodium heparin (UFH), and oversulfated chondroitin sulfate (OSCS) are indicated on the panel.

Pharmaceutical heparins from two manufacturers (three batches of each) were analyzed by classical biochemical methods, including electrophoresis and hexuronic acid, protein and nucleic acid contents, as summarized in Figure 2. We observed the similarities among the six batches, all attending pharmacopeial requirements [26,27,28].

### 3.2. Chromatographic Analysis

**Figure 3:**
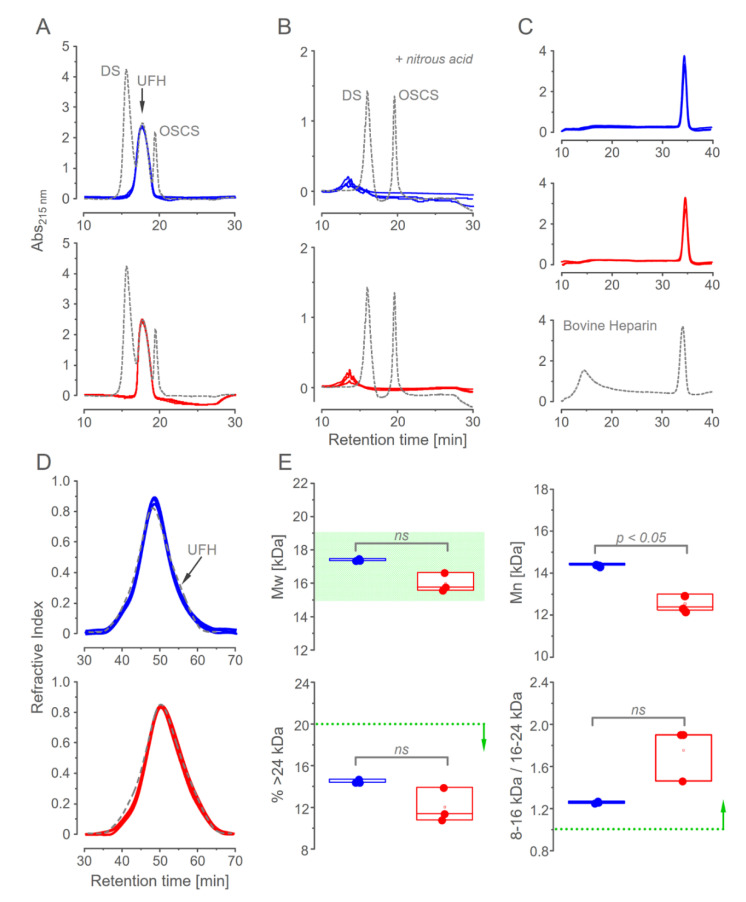
Chromatographic analysis of the pharmaceutical heparins. Ion-exchange chromatography: Overlapped elution profiles of three batches of heparin from each manufacturer 1 (**blue**) and manufacturer 2 (**red**), before and after deamination with nitrous acid, are shown in Panels (**A**,**B**), respectively. Black dotted lines show the elution of the standards of dermatan sulfate (DS), sodium heparin (UFH), and oversulfated chondroitin sulfate (OSCS). Panel (**C**) shows the elution from a strong anion exchange column using step-wise salt gradient of the pharmaceutical heparins in comparison with bovine heparin (**dotted gray line**) for assessing their homogeneities. Panels (**D**,**E**): Size-exclusion chromatography. Overlapped elution profiles of the heparins from manufacturers 1 and 2 and the “Heparin sodium molecular-weight calibrant” (**dotted-black line**). Experimental data were used to calculate the parameters of average molecular mass (M_w_), mean molecular mass (M_n_), and the distribution of heparin molecules by molecular mass ranges, as shown in Panels (**E**). The acceptance criteria established by USP are indicated in green and dotted lines + vertical arrows. *Ns*, non-significant; *p* < 0.05, significant differences; and *t,* test.

Anion exchange and gel permeation chromatography were employed to assess the similarities among the six batches of pharmaceutical heparins. Weighted elution profiles on the anion-exchange chromatography of the three batches from each manufacturer 1 and 2 showed undistinguished profiles (Figure 3A). The analysis was complemented by deamination with nitrous acid, which cleaves heparin but not dermatan sulfate and oversulfated chondroitin sulfate (Figure 3B). It ensures the absence of minor amounts of contaminant dermatan sulfate. Finally, a distinguished salt gradient to elute heparin from the anion-exchange column further assured the homogeneity of the preparation (Figure 3C). Heparin from the bovine intestine shows two components when eluted with this salt gradient [10,12].

Gel permeation chromatography is another essential approach to ensure similarity among pharmaceutical heparins. The molecular size has a significant impact on the anticoagulant activity [29]. The six batches of heparin showed similar profiles of molecular size distribution (Figure 3D). We observed minor but non-significant differences between the heparins from the two suppliers (M_w_ of 17,300 ± 540 vs. 15,996 ± 566 Da for heparins from manufacturers 1 and 2, respectively). In addition to this discrete difference, heparins from the two suppliers attend pharmacopeia recommendations for molecular size distribution (Figure 3E) [26]. Heparins from bovine lung and ovine intestine have distinguished molecular profiles, with the preponderance of chains with a low-molecular size [12].

### 3.3. NMR Spectroscopy

Infrared spectroscopic was a pioneer method for the analysis of heparin and other glycosaminoglycans [30]. Although it is still a useful and reliable technique [31], infrared spectroscopy is rarely used nowadays and has been replaced by NMR methods, which are more sensitive and informative. The combination of 1D and 2D NMR experiments allows access to specific parameters at the atomic level, which enables the exact determination of the monosaccharide composition, sulfation, and *N*-acetylation patterns, linkage positions and anomeric configurations [32,33,34].

**Figure 4:**
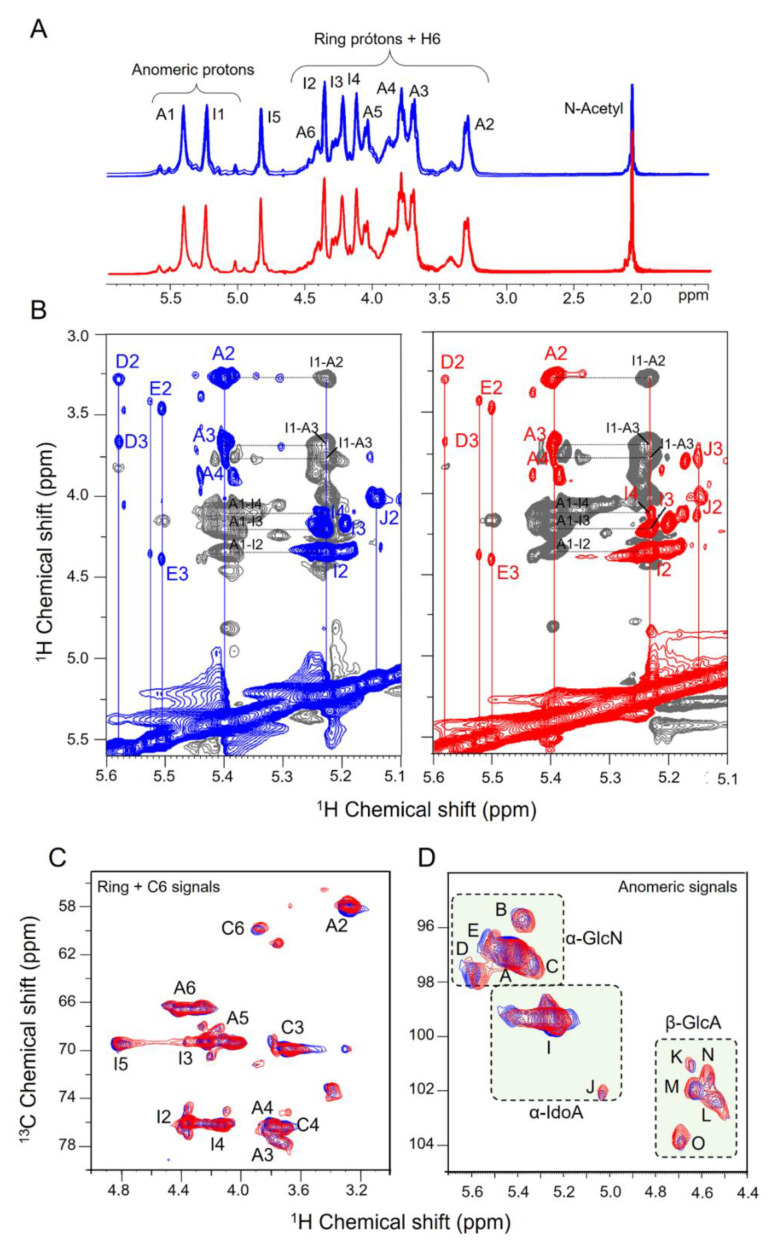
NMR analyses of the pharmaceutical heparins. Panel (**A**): overlapped 1D ^1^H NMR spectra of three batches of heparins from each manufacturer 1 (**blue**) and manufacturer 2 (**red**). Panel (**B**): Representative 2D ^1^H-^1^H phase-sensitive TOCSY spectra of one batch each from the two manufacturers. Panels (**C**,**D**): Overlapped 2D ^1^H-^13^C spectra of one batch each from the two manufacturers. Note that the signals in purple result from the merging of the individual signals in red and blue from each heparin, indicating their similarities. Anomeric signals are assigned and quantified as indicated in Table 3.

**Figure 5:**
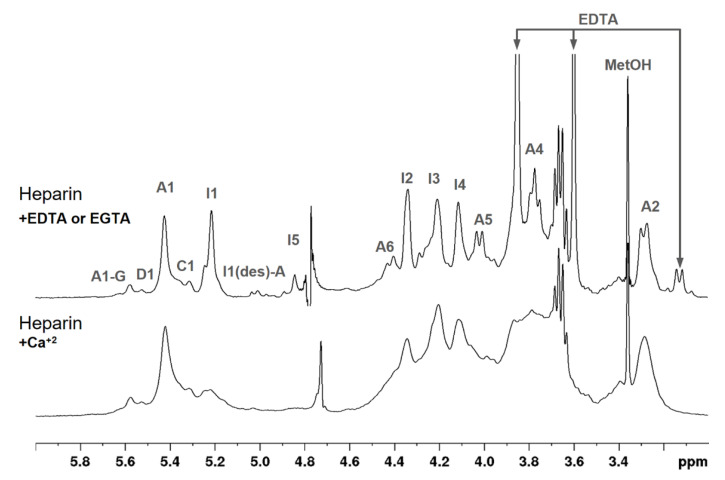
One-dimensional 1H NMR spectra (3.0 → 6.0 ppm) of a pharmaceutical heparin batch containing calcium, before and after the addition of EDTA. Signals attributed to EDTA are indicated with arrows. Note the absence of the I1 signal in the spectrum of heparin containing calcium, which in turn becomes well-defined signals after addition of calcium-chelators in the sample.

Figure 4 summarizes 1D and 2D NMR analysis of the six batches of sodium heparins. One-dimensional proton spectra profiles were very similar, indicating no structural variations among batches or between the two manufacturers (Figure 4A). No additional signals attributed to contaminants were observed on the spectra. Integrals of the major anomeric signals showed no significant variations (Table 2). The well-defined and sharp signals of H1 and H5 of the α-IdoA2S also excluded the occurrence of calcium bound to these heparins. In this case, the signals are less intense and widespread, but reverse to its typical magnitude when EDTA or EGTA is added to the solution (Figure 5).

We conducted further investigations on the pharmaceutical heparins using phase-sensitivity ^1^H-^1^H TOCSY spectra, which unequivocally allow assignments of the protons from the monosaccharide units of heparin. The representative spectra of one batch each from the two manufacturers are shown in Figure 4B. Notably, the spectra acquired from the heparins of both manufacturers demonstrate great similarity, indicating their structural equivalence. Protons signals from the same monosaccharide unit appear in-phase and correlate via scalar coupling (*J*, correlation via chemical bond), as indicated by color of the vertical lines in the panels (blue or red). The anti-phase signals in these spectra are essential for the identification of adjacent units through dipolar coupling (ROE, correlation via spatial proximity), as indicated by the black horizontal-dashed lines. Comparison of the proton chemical shifts obtained from ^1^H-^1^H TOCSY spectra agree with literature data [35].

Finally, NMR analysis was concluded with the basis on 2D ^1^H-^13^C HSQC spectra [36,37]. The overlapping of the two representative spectra from each manufacturer is shown in Figure 4C. The signals in blue from the spectrum of heparin, from manufacturer 1, and in red, from manufacturer 2, emerge in purple due to their precise superimposition. No signals retaining the original color were observed. Clearly, 2D ^1^H-^13^C HSQC spectra assure structure equivalence among those batches of pharmaceutical heparins.

The signals of the 2D ^1^H-^13^C HSQC spectra were assigned according to the literature data [10,14,36,37]. The regions of 3.1/50–5.9/110 (^1^H/^13^C) ppm contain protons/carbons from the pyranose ring + C6 (Figure 4C). Only signals attributed to the major units of heparin were present and completely assigned. These protons/carbons are enumerated on the structure of heparin shown in Figure 1.

The anomeric regions of the spectra are in Figure 4D. We can identify distinct ^1^H-^13^C pairs from different monosaccharide units, as annotated in the spectra. Five pairs are attributed to α-Glc*N* units, two to α-IdoA, and four to β-GlcA residues. These signals from three batches each of heparins from the two manufacturers were quantified by integrating their corresponding signals into the ^1^H-^13^C HSQC spectra (Table 3). The contents of the *N*-sulfation, 3-sulfation, and *N*-acetylation of the α-Glc*N* units and the 2-sulfation of α-IdoA were derived from signals’ integrals (Table 3). However, the content of 6-sulfation is underestimated due to the limitation of the technique. We cannot unequivocally assign the 6-sulfation of signals B and C (α-Glc*N*S units linked to non-sulfated α-IdoA or β-GlcA, respectively). Alternatively, we can calculate the degree of 6-sulfation based on the integrals of H6/C6 signals. The proton/carbon from 6-desulfated α-Glc*N* resonates at ~3.96–59.6 ppm (^1^H-^13^C) while a ~0.6/6.0 downfield shift occurs upon 6-sulfation. These signals, attributed to H6 of 6-sulfated and non-sulfated α-Glc*N* units, are indicated as A6 and C6 in Figure 4C, respectively.

The occurrence of α-Glc*N*,3,6-triS units is in minor amounts and the calculation of their contents require careful interpretation. Furthermore, NMR spectra do not allow to determine the position of this unit along the heparin chain. An alternative is the analysis of oligosaccharides released by heparinases digestion. However, this approach is laborious and difficult to interpret due to the lack of appropriated standards [38,39]. Despite these limitations, we noted only minor differences among the integrals of the pharmaceutical heparins from the two manufacturers. In particular, heparin from manufacturer 2 contains a minor but significantly higher content of *N*-acetylated α-Glc*N* than those from manufacturer 1 (6.6 ± 4.1 vs. 4.1 ± 1.6, respectively).

In summary, we conducted the NMR investigations on six batches of pharmaceutical heparins from two distinct manufacturers in order to determine their monosaccharide composition, sulfation, and acetylation patterns, linkage sites and anomeric configurations. By using phase-sensitive ^1^H-^1^H TOCSY spectra, we were able to unambiguously assign the protons originating from heparin monosaccharide units. The spectra obtained from the heparins from both manufacturers exhibited significant similarity, indicating their structure equivalence. We also utilized 2D ^1^H-^13^C HSQC spectra to identify the signals from different monosaccharide units in the anomeric region and quantify the contents of *N*-sulfation, 6-sulfation, 3-sulfation, and *N*-acetylation of the α-Glc*N* units and 2-sulfation of α-IdoA. Heparins from both manufacturers were found to be structurally similar, with only minor differences in the proportion of *N*-acetylated α-Glc*N*. The use of NMR techniques to analyze heparin and other glycosaminoglycans is crucial for ensuring their quality and safety in pharmaceutical applications [14,32,33,34,36,37].

### 3.4. Analysis Based on Digestion with Heparinases

A comparative study among pharmaceutical heparins requires a combination of different methodologies to meticulously address their complex chemical structures. We complemented the spectroscopic analysis using assays based on digestion with heparinases. Isomeric hexuronic acids (α-IdoA and β-GlcA) cannot be identified by this method because heparinases are eliminated, introducing unsaturation into non-reducing terminals between positions 4 and 5. The same type of unsaturated hexuronic acid is obtained from both α-IdoA and β -GlcA. This methodology also does not allow the identification of disaccharides containing α-Glc*N*,3,6-triS, which are not cleaved by heparinases. Besides these limitations, the enzymatic method permits a more accurate determination of the pattern of *N*-acetylation and *N*-sulfation of the α-Glc*N*. It also assures a more precise way to assess the 6-sulfated α-Glc*N* units, which is a limitation of the 2D ^1^H-^13^C HSQC spectra [40].

**Figure 6:**
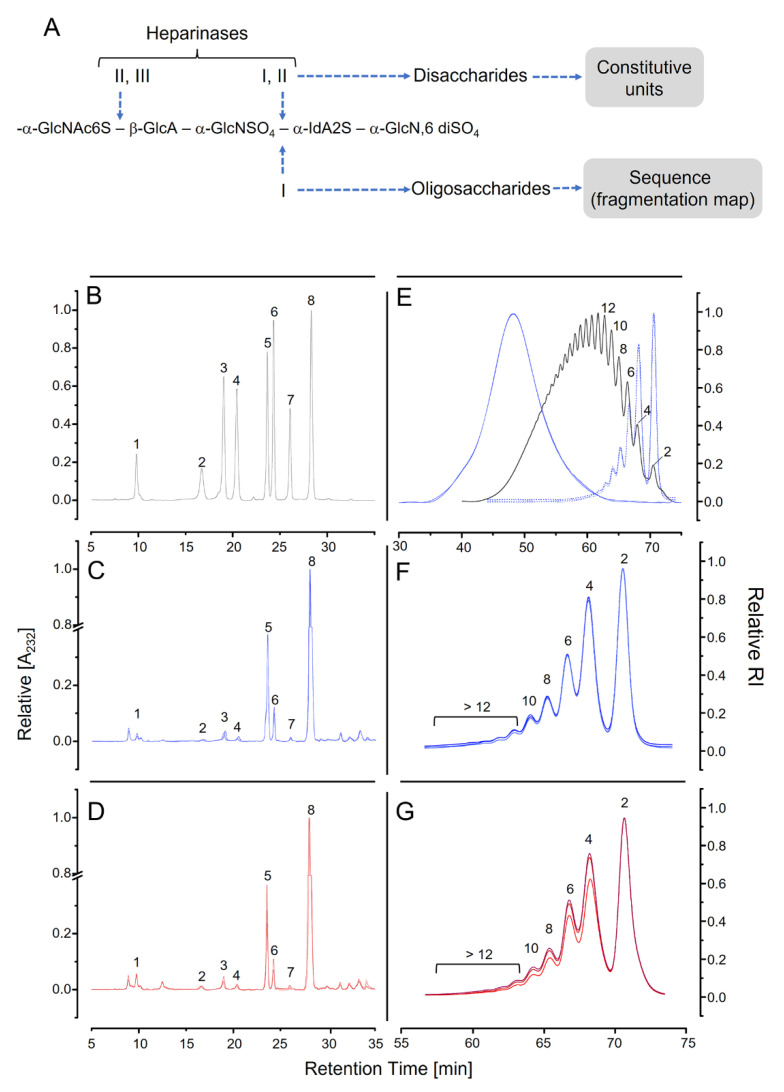
Analyses of pharmaceutical heparins by digestions with heparinases. Panel (**A**): Summary of the experimental approach employed in the analyses. Panels (**B**–**D**): Chromatograms of the disaccharides formed by the digestion of heparins with a mixture of heparinases I, II, and III. Elution of standard disaccharides is on Panel (**B**) (**black**) and enumerated as in Table 4. Panels (**C**,**D**) show overlapping of the elution profiles of the disaccharides formed from 3 heparin batches from each manufacturer 1 (**blue**) and manufacturer 2 (**red**) by digestion with the heparinases. Panels (**E**–**G**): Size-exclusion chromatograms of the oligosaccharides formed after the digestion of heparins with heparinase I. Panel (**E**) shows the chromatograms of non-digested heparins (**dashed line in blue**), three batches from manufacturer 1, and the oligosaccharides formed by the action of the enzyme (in blue). The black-dotted line shows the elution of the “International standard of low-molecular-weight heparin for molecular weight calibration”. Numbers in the panel indicate the degree of polymerization of the oligosaccharides. Panels (**F**,**G**) show an expansion of the chromatograms (fractions 55 → 76) of the products formed after the digestion of heparins from the two manufacturers by heparinase I.

Two types of approaches were employed using these enzymes (Figure 6A). One involves the simultaneous digestion of heparin with a mixture of heparinases I, II, and III. Disaccharides with different sulfation and *N*-acetylation patterns are released. They were separated by ion-exchange chromatography and compared with standards, which allows their identification and quantification. These results enable the calculation of the proportions of the constitutive units of heparin. Another approach is based on the use of a single enzyme (heparinase I), which acts exclusively on the glycoside-bonds containing α-IdoA. The bonds formed by β-GlcA are resistant to this enzyme. As a consequence, different oligosaccharides are formed, according to the distribution of these two hexuronic acids along the heparin chain. The oligosaccharides released by the enzyme yields characteristic fragmentation maps for each type of heparin [41].

Chromatograms of the products formed after cleaving the six batches of heparins with heparinases are shown in Figure 6C,D. The elution of standard disaccharides is shown in Figure 6B. The main product formed (~75%) is the tri-sulfated disaccharide (ΔUA2S-Glc*N*S,6S; Table 4), as expected. It corresponds to units A-a on the 2D ^1^H-^13^C HSQC spectra. Based on the proportions of the disaccharides formed by the heparinases, we can calculate the content of 2-sulfation, 6-sulfation, *N*-sulfation, and *N*-acetylation, as indicated in Table 4. We observed minute differences among the batches from the same manufacturer. More expressive are the significant differences in the content of *N*-acetylated α-Glc*N* observed between the heparins from the two manufacturers (5.6 ± 0.70 vs. 8.5 ± 0.47, manufacturers 1 and 2, respectively). This result agrees with the data from the ^1^H-^13^C HSQC spectra (Table 3). We also observed significant differences between the content of 6-sulfation units between heparins from the two manufacturers but this observation was not confirmed by the NMR data.

Subsequently, we digested the pharmaceutical heparins with heparinase I and analyzed the oligosaccharides formed by gel permeation chromatography. A typical assay shows heparin before and after digestion with heparinase I (Figure 6E, in blue). The degree of polymerization of the oligosaccharides was determined by comparing with the elution profile of a molecular-mass standard of low-molecular-weight heparin (Figure 6E, in black). The products formed by the enzyme are predominantly di- and tetrasaccharides, >60% of the total. The oligosaccharides formed by the heparinase I digestion of the heparin from the two manufactures (Figure 6F,G) show similar fragmentation maps, denoting the equivalent distribution of β-GlcA units in the heparin chains.

### 3.5. Fluorescence Spectroscopy

A complex aspect of the comparability studies among heparins is to assure the similar content of structural components present in minor quantities but with significant biological relevance. Heparin has some regions made up of a specific pentasaccharide sequence with high-affinity with antithrombin. This minor component is essential for the anticoagulant activity [42]. The most expressive structural component of this region is the α-Glc*N*,3,6-triS units. The small quantities of this unit make it very difficult to quantify using 2D ^1^H-^13^C HSQC spectra (Table 3). They are also not detected after heparin digestion with heparinases since α-Glc*N*,3,6-triS units are not cleaved by these enzymes [14]. An alternative is assessing the interactions of heparin with antithrombin by fluorescence spectroscopy [25,43,44]. This is an indirect method to detect the content of α-Glc*N*,3,6-triS units.

**Figure 7:**
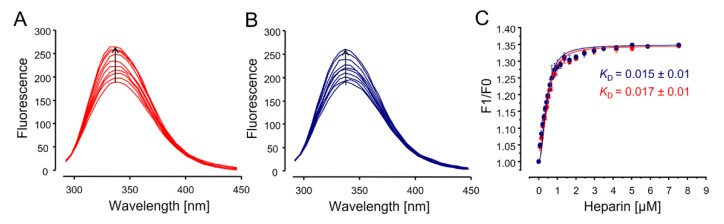
Fluorescence analyses to determine the interactions of pharmaceutical heparins with antithrombin. Panels (**A**,**B**) show the gain in antithrombin fluorescence promoted by the binding with heparins from manufacturers 1 (**blue**) and 2 (**red**). Titration curves derived from these data (as the mean ± SD of three batches from each manufacturer) shown in Panel (**C**) were used to calculate the dissociation constants (*K*_D_) for the interaction of heparin with antithrombin, as indicated in the panel.

Figure 7A,B show the increase in the intrinsic fluorescence of antithrombin as heparin is added to the solution. These data allow obtaining the titrating curve for the interaction between the two molecules. Batches of heparin from the two manufacturers showed similar titration curves, with the same amplitude and maximum increase in intensity and at the same wavelength (Figure 7C). The dissociation constants (*K*_D_) calculated for the interactions of antithrombin with the six batches of pharmaceutical heparins are ~0.01 µM, significantly lower than observed for heparins from other source, such as bovine heparins (~0.28 µM) [10]. Therefore, heparins from the two manufacturers indicated by the fluorescence spectroscopy to have similar contents of pentasaccharide with high affinity for antithrombin. Units of α-Glc*N*,3,6-triS are representative components of this region.

### 3.6. Anticoagulant Activity

**Figure 8:**
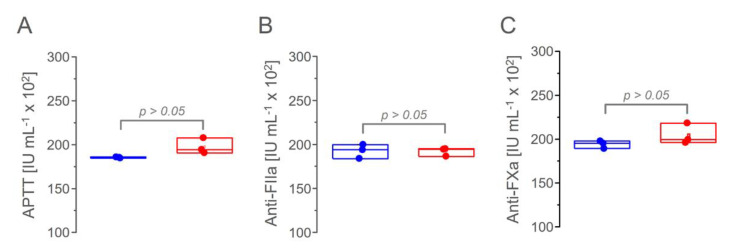
Anticoagulant activity of the pharmaceutical ampules formulated with heparin from manufacturers 1 (**blue**) and 2 (**red**) based on APTT (Panel (**A**)), anti-FIIa activity (Panel (**B**)) and anti-FXa activity (Panel (**C**)) assays.

Finally, we determined the anticoagulant activity of the six batches of pharmaceutical heparins based on the APTT, anti-FIIa, and anti-FXa assays (Figure 8). We did not observe significant differences among the six batches or between the products from the two manufacturers. Thus, the minor differences in the physicochemical properties of the heparins from manufacturers 1 and 2 was mainly their constitutive units and molecular sizes, which did not impact the anticoagulant activity [14].

## 4. Conclusions

Pharmaceutical heparins are regularly analyzed following pharmacopeial protocols. In this study, we evaluated the batches obtained from two manufacturers, formulated with APIs from China or Brazil, using more refined analytical methods, which are significantly ahead from those of the pharmacopeias. We combined the methods focusing on different properties of heparin as well as evaluated a single aspect with distinct approaches (Table 1). Thus, chromatographic methods check the molecular size and ionic properties. The absence of contaminants is verified by biochemical methods but confirmed in further detail by NMR spectroscopy. The monosaccharide composition, sulfation and *N*-acetylation patterns, linkage position, and anomeric configuration were verified by a combination of enzymatic methods and NMR spectroscopy. Minor components, especially the tri-sulfated α-Glc*N*, was also evaluated by an indirect approach based on fluorescence spectroscopy. As such, we can verify the similarities or dissimilarities among the major and minor components of heparin with a high degree of accuracy. Finally, the anticoagulant activity was verified by three methods: a clotting assay and two tests based on purified proteins (anti-FIIa and anti-FXa activities).

The results obtained showed minimal differences among the constitutive disaccharides units that make up the heparins obtained from the two manufacturers. These results were assured by integrals derived from the 2D ^1^H-^13^C HSQC spectra and the quantification of the disaccharides formed by heparinases digestion. The two manufacturers’ heparins differ in their contents of *N*-acetylated α-Glc*N*. They also differ in molecular mass distribution, as indicated by gel permeation chromatography. These differences in the physicochemical properties of the pharmaceutical heparins have no impact on their anticoagulant activity. Possibly, they indicate the particularities of distinguishing manufacturers but with no impact on biological activity and clinical use.

Currently, comparability studies to establish the sameness between different heparin products, *viz*. pharmaceutical formulations and APIs, are mostly carried out on the basis of compendial assays from the heparin monographs of main pharmacopeias [26,27,28]. We recently demonstrated that even minor differences between heparins from different animal sources could impact biological effects [14], and thus, eventual microheterogeneities among products from different manufacturers should be detected in minor details to avoid inconsistencies in their anticoagulant activities [45]. The analytical framework proposed here including both compendial and state-of-the art assays has proven to be robust enough to assure the sameness of different heparins, and thus could be used as a basis for regulatory agencies to establish new guidelines for comparing different products, such as those made available by the FDA to compare enoxaparins [21].

Heparin is an essential and life-saving drug in medicine. There are no other drugs in development for several of its clinical uses. We also have no perspective on how heparin may be obtained by chemical or enzymatic synthesis, especially in the quantities needed to meet the present demand, which is at the level of tons. The preparation of heparin exclusively from pig intestine cannot meet the increased demand [46,47]. The alternative is to use other animal sources to obtain pharmaceutical heparins, such as bovine and ovine intestines. However, these preparations have significant structural and biological differences [14]. To address this issue, heparins from different sources can be identified as distinct drugs. This is the alternative that has already become implemented in Brazil, with separate monographs for porcine and bovine heparins [6].

However, we still need to address the approaches to evaluate pharmaceutical preparations from the same animal source, but prepared by distinct manufacturers and/or in different countries. Obviously, clinical tests are needed to ensure the safety, efficacy, and similarity of this diversity of pharmaceutical heparins. However, these studies are costly and time-consuming. They can be abbreviated by a similarity assessment based on the protocols of physicochemical techniques and biological assays. This was a strategy successfully followed for low-molecular-weight heparins, which we propose to extend to unfractionated heparin [20,21].

A final comment that deserves consideration is that the demonstration of “dissimilarity” is an easy approach in science. Evidence that two molecules have slight differences in their structure or activity is enough to assure that they are “different”. In contrast, the demonstration of “similarity” is an almost endless task, especially in the case of complex molecules such as heparin. In practical terms, decisions regarding its clinical use will depend on a variety of aspects, including cost, similarity in physicochemical and biological assays and, finally, clinical tests.

## Figures and Tables

**Table 1:** Criteria and assays (compendial and in-depth approaches) proposed for performing comparability studies of pharmaceutical heparins.

Criterium	Compendial Assays	In-Depth Approaches
(1) Absence of contaminants	Biochemical assays IEX-HPLC 1D ^1^H-NMR	IEX-HPLC/nitrous acid deamination
(2) Equivalence of molecular mass	SEC-HPLC	-
(3) Animal source sameness	IEX-HPLC 1D ^1^H-NMR	Disaccharide analysis/IEX-HPLC/heparinases Fragmentation map/SEC-HPLC/heparinase I
(4) Exclude mixtures of heparins from different animal sources	1D ^1^H-NMR	2D ^1^H-^13^C HSQC NMR Disaccharide analysis/IEX-HPLC/heparinases
(5) Equivalence of the disaccharide composition	-	2D ^1^H-^13^C HSQC NMR Disaccharide analysis/IEX-HPLC/heparinases
(6) Equivalence of minor components	-	2D ^1^H-^13^C HSQC NMR 2D ^1^H-^1^H TOCSY NMR Disaccharide analysis/IEX-HPLC/heparinases Affinity heparin-AT/Fluorescence spectroscopy
(7) Equivalence in the sequence of residues	-	2D ^1^H-^13^C HSQC NMR 2D ^1^H-^1^H TOCSY NMR Fragmentation map/SEC-HPLC/heparinase I
(8) Equivalence in biological assays	Anti-FXa activity assay Anti-FIIa activity assay	Fluorescence spectroscopy/Affinity heparin-AT

IEX-HPLC: Ion-exchange chromatography. SEC-HPLC: Size-exclusion chromatography. NMR: Nuclear magnetic resonance.

**Table 2:** Integrals of the anomeric signals of the six batches of heparin from two manufactures.

Heparin	Batch	Signal (Structure) ^1^			
		A1 (α-GlcN,6-diS)	C1 (α-GlcNS)	I1 (α-IdoA2S)	C1 × 100/A1 ^2^
Manufaturer 1	1	1.00	0.09	0.83	9
	2	1.00	0.10	0.82	10
	3	1.00	0.10	0.83	10
	Mean ± SD ^3^	1 ± 0	0.09 ± 0.005	0.83 ± 0.005	10 ± 0.5
Manufacturer 2	4	1.00	0.11	0.80	11
	5	1.00	0.10	0.81	10
	6	1.00	0.10	0.84	10
	Mean ± DP ^3^	1 ± 0	0.10 ± 0.005	0.82 ± 0.02	10 ± 0.5

^1^ A1, C1, and I1 are the anomeric signals of α-GlcN,6-diS, α-GlcNS, and α-IdoA2S, respectively. See [14] for further details. ^2^ Calculation recommended by the Brazilian Pharmacopeia. ^3^ Non-significant differences (*p* > 0.05), *t* test.

**Table 3:** Quantification of the monosaccharide units (%) of pharmaceutical heparin from two manufacturers calculated based ^1^H-^13^C HSQC spectra.

	Unit ^1^	Proportion %							
		Manufacturer 1				Manufacturer 2			
		1	2	3	$\bar{X}$ ± dp	4	5	6	$\bar{X}$ ± dp
α-Glc*N*:									
E	α-Glc*N*,3,6-triS-(?)	5.2	5.1	5.4	5.2 ± 0.2	5.7	5.5	5.6	5.6 ± 0.1
A-a	α-Glc*N*,6-diS-(α-IdoA2S)	66.5	66.1	61.5	64.7 ± 2.8	60.4	61.7	62.2	61.4 ± 0.9
A-b	α-Glc*N*Ac-(β-GlcA)	**3.8**	**4.2**	**4.3**	**4.1 ± 0.3**	**6.1**	**6.1**	**7.5**	**6.6 ± 0.8**
B	α-Glc*N*S-(α-IdoA)	6.9	4.9	7.1	6.3 ± 1.2	8.2	7.0	9.2	8.1 ± 1.1
D	α-Glc*N*S-(β-GlcA)	9.1	11.3	14.0	11.5 ± 2.4	10.6	12.6	7.5	10.2 ± 2.6
C	α-Glc*N*S-(α-IdoA2S)	8.6	8.4	7.8	8.3 ± 0.4	9.0	7.0	8.0	8.0 ± 1.0
	∑ α-Glc*N*	100	100	100	100.0	100	100	100	100.0
α-IdoA:									
I	α-IdoA2S	79.4	77.9	81.0	79.4 ± 1.6	77.2	76.6	77.6	77.1 ± 0.5
J	α-IdoA-(Glc*N*,6S)	2.1	3.2	3.0	2.8 ± 0.6	1.6	2.1	1.6	1.8 ± 0.3
-	α-IdoA	1.5	0.9	1.1	1.2 ± 0.3	1.0	1.4	1.1	1.2 ± 0.2
	∑ α-IdoA	83.0	82.1	85.1	83.4 ± 1.5	79.8	80.0	80.3	80.0 ± 0.3
β-GlcA:									
K	β-GlcA-(α-Glc*N*,3,6-triS)	**3.0**	**2.4**	**2.6**	**2.7 ± 0.3**	**1.4**	**1.2**	**2.1**	**1.6 ± 0.5**
L	β-GlcA-(α-Glc*N*Ac)	**6.9**	**8.0**	**4.7**	**6.5 ± 1.7**	**11.4**	**11.1**	**10.6**	**11.0 ± 0.4**
M	β-GlcA-(α-Glc*N*S)	7.1	7.5	7.7	7.4 ± 0.3	7.5	7.7	7.0	7.4 ± 0.4
	∑ β-GlcA	17.0	17.9	14.9	16.6 ± 1.5	20.2	20.0	19.7	20.0 ± 0.3
α-Glc*N*	*N*-sulfatation ^2^	**96.3**	**95.8**	**95.7**	**95.9 ± 0.3**	**93.9**	**93.9**	**92.5**	**93.4 ± 0.8**
	6-Sulfatation ^3^	87.4	86.2	84.1	85.9 ± 1.7	86.3	84.6	85	85.0 ± 0.9
	3-Sulfatation ^2^	5.2	5.1	5.4	5.2 ± 0.2	5.7	5.5	5.6	5.6 ± 0.1
	*N*-Acetylation ^2^	**3.8**	**4.2**	**4.3**	**4.1 ± 1.6**	**6.1**	**6.1**	**7.5**	**6.6 ± 0.5**
α-IdoA	2-Sulfatation ^2^	79.4	77.9	81.0	79.4 ± 0.3	77.2	76.6	77.6	77.1 ± 0.8

^1^ Units in brackets are subsequent residues. ^2^ Calculated using integrals of the anomeric signal in Figure 4D. ^3^ Calculated based on the H6/C6 integrals of 6-sulfated and non-sulfated α-Glc*N*. See signals A6 and C6, respectively, in Figure 4C. Values in bold indicate significant differences *p* < 0.05 (*t* test).

**Table 4:** Proportions (% of total) of the disaccharides formed by the digestion of pharmaceutical heparins from two manufacturers with a mixture of heparinases I, II, and III.

Disaccharides ^1^	Manufacturer 1				Manufacturer 2			
	1	2	3	Mean ± SD	4	5	6	Mean ± SD
1: ΔUA-Glc*N*Ac	**1.7**	**1.1**	**1.0**	**1.2 ± 0.39**	**4.0**	**3.4**	**3.1**	**3.5 ± 0.45**
2: ΔUA-Glc*N*S	**0.7**	**0.6**	**0.7**	**0.6 ± 0.07**	**1.7**	**1.4**	**1.3**	**1.5 ± 0.22**
3: ΔUA-Glc*N*Ac,6S	2.5	2.4	2.6	2.5 ± 0.09	2.8	2.7	3.0	2.8 ± 0.17
4: ΔUA2S-Glc*N*Ac	1.3	0.8	1.1	1.0 ± 0.23	1.3	1.4	1.3	1.4 ± 0.06
5: ΔUA-Glc*N*S,6S	18.5	18.7	18.8	18.7 ± 0.12	13.8	11.8	15.8	13.8 ± 2.00
6: ΔUA2S-Glc*N*S	5.1	4.6	4.7	4.8 ± 0.29	3.7	3.4	4.6	3.9 ± 0.65
7: ΔUA2S-Glc*N*Ac,6S	0.9	0.7	0.7	0.8 ± 0.08	0.9	0.6	1.1	0.9 ± 0.27
8: ΔUA2S-Glc*N*S,6S	69.3	71.1	70.5	70.5 ± 0.94	71.8	75.3	69.7	72.3 ± 2.82
2-sulfation	76.5	77.2	77.0	76.9 ± 0.35	77.7	80.7	76.8	78.4 ± 2.05
6-sulfation	**91.2**	**92.9**	**92.6**	**92.3 ± 0.91**	**89.3**	**90.4**	**89.7**	**89.7 ± 0.53**
*N*-sulfation	**93.7**	**95.0**	**97.1**	**94.7 ± 0.70**	**90.9**	**91.9**	**91.5**	**91.4 ± 0.47**
*N*-acetylation	**6.3**	**5.0**	**5.3**	**5.6 ± 0.70**	**9.1**	**8.1**	**8.5**	**8.6 ± 0.47**

^1^ Numbers used to identify the disaccharides on the chromatograms of Figure 6B–D. Values in bold indicate significant differences *p* < 0.05 (*t* test).
